# Grapevine cell early activation of specific responses to DIMEB, a resveratrol elicitor

**DOI:** 10.1186/1471-2164-10-363

**Published:** 2009-08-06

**Authors:** Anita Zamboni, Pamela Gatto, Alessandro Cestaro, Stefania Pilati, Roberto Viola, Fulvio Mattivi, Claudio Moser, Riccardo Velasco

**Affiliations:** 1 IASMA Research and Innovation Center, Fondazione Edmund Mach, S. Michele a/Adige I-38010, Italy; 2 Current address: Department for Sciences, Technologies and Markets of Grapevine and Wine, Via della Pieve 70, I-37029 San Floriano di Valpolicella (VR), Italy; 3 Current address: Centre for Integrative Biology (CIBIO), University of Trento, Via delle Regole 101, 38060 Mattarello (TN), Italy

## Abstract

**Background:**

In response to pathogen attack, grapevine synthesizes phytoalexins belonging to the family of stilbenes. Grapevine cell cultures represent a good model system for studying the basic mechanisms of plant response to biotic and abiotic elicitors. Among these, modified *β*-cyclodextrins seem to act as true elicitors inducing strong production of the stilbene resveratrol.

**Results:**

The transcriptome changes of *Vitis riparia *× *Vitis berlandieri *grapevine cells in response to the modified *β*-cyclodextrin, DIMEB, were analyzed 2 and 6 h after treatment using a suppression subtractive hybridization experiment and a microarray analysis respectively. At both time points, we identified a specific set of induced genes belonging to the general phenylpropanoid metabolism, including stilbenes and hydroxycinnamates, and to defence proteins such as PR proteins and chitinases. At 6 h we also observed a down-regulation of the genes involved in cell division and cell-wall loosening.

**Conclusions:**

We report the first large-scale study of the molecular effects of DIMEB, a resveratrol inducer, on grapevine cell cultures. This molecule seems to mimic a defence elicitor which enhances the physical barriers of the cell, stops cell division and induces phytoalexin synthesis.

## Background

Plants respond to pathogens through constitutive and inducible mechanisms [1]. Structural barriers represent preformed constitutive defences, while the accumulation of pathogenesis-related proteins (PR), phytoalexins and reactive oxygen species is part of an active mechanism stimulated by the pathogen [2]. Grapevine also responds to fungal infection via PR-protein synthesis and phytoalexin accumulation [3]. Plant phytoalexins are low-molecular-weight secondary metabolites with antimicrobial properties and they show wide chemical diversity among different plant species [4]. In grapevine they mainly belong to the stilbene family and consist of *trans*-resveratrol (3,5,4'-trihydroxystilbene) its oligomers, called viniferins [5-7] and pterostilbene, a dimethylated derivative of resveratrol [8]. Stilbene synthesis in berries [9] and leaves can be elicited by fungal infection [5,10], but also by treatment with UV-irradiation [11], ozone [12] and heavy metals [13].

Plant cell cultures are a useful tool for studying plant cell defence response to biotic and abiotic elicitors [14]. Stilbene accumulation has been reported in grapevine cells treated with different elicitors: fungal cell wall fragments [15], Na-orthovanadate, jasmonic acid and methyljasmonate [16,17] and laminarin, a *β*-glucan polysaccharide from brown algae [18]. In addition, special attention has been given to the *β*-cyclodextrin molecular class. These are cyclic oligosaccharides consisting of seven *α*-D-glucopyranose residues linked by *α *1 → 4 glucosidic bonds forming a structure with a hydrophobic central cavity and a hydrophilic external surface [19]. Among *β*-cyclodextrins, heptakis(2,6-di-*O*-methyl)-*β*-cyclodextrin (DIMEB), was reported to be the most effective resveratrol elicitor in different *Vitis vinifera *cultivars [19,20]. The ability of the modified *β*-cyclodextrins to act as elicitors probably resides in their chemical similarity to the alkyl-derivatized pectic oligosaccharides released from the cell walls during fungal infection [20]. Along with stilbene accumulation these experiments highlighted a more general response involving peroxidase activity as well as inhibition of *Botrytis cinerea *growth [19,20].

Zamboni et al. [21] further investigated DIMEB activity on additional *Vitis *genotypes and observed that its effect was more pronounced when tested on *Vitis riparia *× *Vitis berlandieri *cell cultures. The kinetics of resveratrol synthesis showed that trans-resveratrol, the induced form, started to accumulate from 6 h after treatment and reached its maximum at 24 h. Moreover, this metabolite was much more localized in the medium than within the cell.

With these results [21] as our starting point, we report here the first large-scale transcriptional characterization of the early response of *Vitis riparia *× *Vitis berlandieri *cells to DIMEB treatment.

After 2 h, 127 positively modulated genes were identified by suppression subtractive hybridization (SSH), whereas after 6 h, 371 genes turned out to be differentially expressed when control and treated cells on the *Vitis vinifera *GeneChip^® ^Genome Array (Affymetrix) were compared. These results showed that DIMEB specifically modulates the expression of a small number of genes involved in resveratrol and lignin biosynthesis, PR synthesis, cell division and cell wall modification.

## Results and discussion

The ability of DIMEB to elicit defence responses in grapevine cell culture was suggested by previous results showing stilbene accumulation, changes in peroxidase activity, as well as inhibition of *Botrytis cinerea *growth [19,20]. Considerable stilbene accumulation in response to DIMEB treatment was also observed by our group using non-*vinifera *(*Vitis riparia *× *Vitis berlandieri*) liquid cell cultures [21]. In this study we analyzed the changes in gene expression of these cells elicited with DIMEB after 2 h and 6 h using SSH and microarray experiments, respectively.

The rationale behind the two approaches was that after 2 h of treatment, a small number of genes are expected to be modulated, and only to a limited extent, whereas after 6 h an increase in the number of genes and in their expression level is envisaged. The SSH technique appeared then the right choice for identifying the low abundance differential transcripts at 2 h, while the Affymetrix GeneChip^® ^microarray was used to measure the expression of a larger number of genes (~14,500 unigenes) after 6 h of treatment [22].

Starting with 384 clones from the constructed cDNA subtractive library and then performing a hybridization screening to eliminate clones which were not really differentially expressed (false positives), we obtained 168 high-quality sequences which clustered in 127 tentative consensuses (Additional File 1). The microarray experiments instead identified 371 (223 upregulated and 148 downregulated) significantly modulated probe sets in the treated cells compared with the control ones (Additional File 2). Sequence annotation and classification according to Gene Ontology categories [23], revealed that at both time points primary (mainly signal transduction related genes) and secondary metabolisms, together with response to the stimulus, were the most affected categories (Additional Files 3 and 4). At 6 h, the analysis also highlighted downregulation of the cellular component organization and the biogenesis category (Additional file 4).

**Figure 1:**
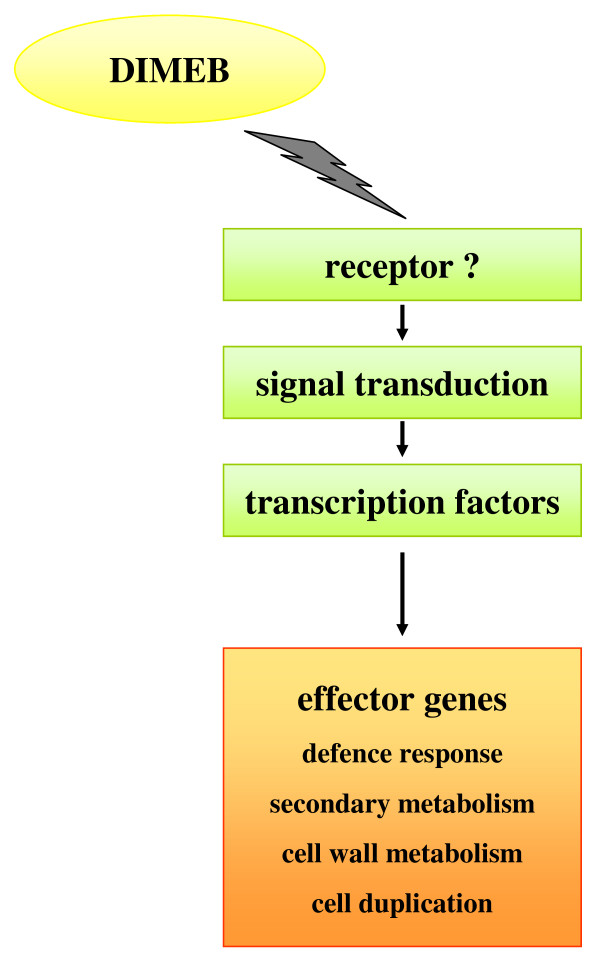
**Molecular events triggered by DIMEB as deduced by transcriptional profiling**.

In general, the two experiments showed modulation of specific mechanisms had already occurred at 2 h and continued more extensively at 6 h after DIMEB treatment. The data summarized in Table 1 suggest that the grapevine cell responds to the elicitor by the activation of a signal transduction cascade which leads to the induction of specific classes of transcription factors. The downstream effect of this process is, on the one hand, the induction of some branches of the secondary metabolism and defence response, and, on the other hand, the blockage of cell duplication (Figure 1).

**Table 1:** List of transcripts modulated by DIMEB and reported in the Discussion

**ID^a^**	**Description**	**Uniprot ID^b^**	**TC-ID^c^**	**2 h**	**6 h**	
					
				**+**	**+**	**-**
**Signal trasduction**						
CLU090	Kinase associated protein phosphatase	P46014	EC987592	x		
1608981_at	Putative phospholipase	Q8RXN7	TC69626		x	
1620080_at	Putative receptor-like protein kinase ARK1	Q5ZAK8	CB922377		x	
1611172_at	SOS2-like protein kinase	Q8LK24	TC52484		x	
**Transcription factors**						
1619311_at	Pathogenesis-related genes transcriptional activator PTI5	O04681	TC55556		x	
1611285_s_at	Probable WRKY transcription factor 11	Q9SV15	TC65678		x	
CLU059	TGA10 transcription factor	Q52MZ2	TC99087	x		
1610775_s_at	WRKY transcription factor-b	Q5DJU0	TC55553		x	
**Effector genes**						
*Phe biosynthesis*						
CLU083	3-Deoxy-D-arabino-heptulosonate 7-phosphate synthase precursor	O24051	TC74975	x		
1611211_at	3-Deoxy-D-arabino-heptulosonate 7-phosphate synthase precursor	O24046	TC57386		x	
1614440_at	3-Deoxy-D-arabino-heptulosonate 7-phosphate synthase	Q6YH16	TC54321		x	
1619357_at	3-Deoxy-D-arabino-heptulosonate 7-phosphate synthase	O24046	TC57642		x	
1621405_at	Plastidic 3-deoxy-D-arabino-heptulosonate 7-phosphate synthase 2	O22407	TC51974		x	
1609646_at	3-Dehydroquinate synthase-like protein	Q9FKX0	TC56854		x	
1609932_at	Prephenate dehydratase	Q6JJ29	TC53641		x	
1621307_at	Prephenate dehydratase	Q6JJ29	TC53641		x	
1611895_at	Putative chorismate mutase	Q5JN19	TC62307		x	
*General phenylpropanoid metabolism*						
1613113_at	Phenylalanine ammonia lyase	Q6UD65	TC60180			
CLU024	Trans-cinnamate 4-monooxygenase	Q43240	TC71512	x		
1610821_at	Cinnamic acid 4-hydroxylase	Q948S8	TC70715		x	
1616191_s_at	Cinnamic acid 4-hydroxylase	Q948S8	TC70715		x	
1615801_at	4-Coumarate:CoA ligase	Q5S017	TC60943		x	
1619320_at	4-Coumarate--CoA ligase 2	P31687	TC66743		x	
*Stilbene biosynthesis*						
CLU009	Stilbene synthase	Q9SPW2	TC89701	x		
CLU022	Stilbene synthase	Q6BAU9	TC89632	x		
CLU023	Stilbene synthase	P28343	TC84974	x		
CLU049	Stilbene synthase	Q8LPP4	TC78210	x		
CLU097	Stilbene synthase	Q9S982	TC84974	x		
CLU103	Stilbene synthase	P28343	TC88894	x		
1606750_at	Stilbene synthase	Q6BAL2	TC67020		x	
1608009_s_at	Stilbene synthase	P51070			x	
1609696_x_at	Stilbene synthase	P28343	TC67020		x	
1609697_at	Stilbene synthase	Q944W7	TC60946		x	
1610824_s_at	Stilbene synthase	Q93YX5	TC52746		x	
1610850_at	Stilbene synthase	P28343			x	
1611190_s_at	Resveratrol synthase	Q94G58	TC67020		x	
1612804_at	Stilbene synthase	Q9SPW2	TC52746		x	
1614621_at	Stilbene synthase	P28343	TC67020		x	
1616575_at	Stilbene synthase	Q944W7	TC52746		x	
1620964_s_at	Stilbene synthase	P28343			x	
1622638_x_at	Stilbene synthase	Q9SPW2	TC52746		x	
*Secondary metabolite transport*						
CLU106	PDR-like ABC transporter	Q8GU88	TC76318	x		
CLU119	Pleiotropic drug resistance protein 12	Q5Z9S8	TC81892	x		
1613763_at	ABC transporter-like protein	Q9LYS2	TC60768		x	
1618493_s_at	ABC transporter-like protein	Q9LYS2	TC64210		x	
1610363_at	CjMDR1	Q94IH6	TC69843		x	
1609330_at	Glutathione S-transferase	Q6YEY5	NP864091		x	
1611890_at	Glutathione S-transferase GST 14	Q9FQE4	TC61062		x	
1619682_x_at	Caffeic acid O-methyltransferase	Q9M560	TC52364		x	
1620342_at	Caffeic acid 3-O-methyltransferase 1	Q00763	TC64352		x	
*Lignin biosynthesis*						
1611897_s_at	Caffeoyl-CoA O-methyltransferase	Q8H9B6	TC63685		x	
1614643_at	Caffeoyl-CoA O-methyltransferase	Q43237	TC51729		x	
1613900_at	Cinnamyl alcohol dehydrogenase	Q9ATW1	TC52904		x	
1614045_at	Ferulate 5-hydroxylase	Q6IV45	TC64493		x	
1614502_at	Ferulate 5-hydroxylase	Q6IV45	TC63764		x	
1619065_at	Putative cinnamoyl-CoA reductase	Q8W3H0	TC53437		x	
1622651_at	Polyphenol oxidase	Q68NI4	TC58764		x	
1610806_at	Putative diphenol oxidase	Q6Z8L2	CD007812		x	
CLU122	Chalcone-flavonone isomerase	P51117	TC78712	x		
CLU048	Flavonol 3-O-glucosyltransferase 6	Q40288	TC85607	x		
1621051_at	Flavonol 3-O-glucosyltransferase 2	Q40285	CN006197			x
*Defence response*						
CLU088	Chitinase (Class II)	Q43322	TC95665	x		
1613871_at	Class IV chitinase	Q9M2U5	TC57889		x	
1617192_at	Class IV chitinase	Q7XB39	TC63731		x	
1617430_s_at	Basic endochitinase precursor	P51613	TC51704		x	
CLU001	Pathogenesis-related protein10	Q9FS42	TC72098	x		
1610011_s_at	Pathogenesis-related protein10	Q9FS42			x	
1618568_s_at	Pathogenesis-related protein10	Q9FS42			x	
CLU021	Pathogenesis-related protein PR-4A precursor	P29062	TC91296	x		
CLU036	Merlot proline-rich protein 2	Q6QGY1	TC85591	x		
1609875_at	Protease inhibitor	Q6YEY6			x	
1611666_s_at	Protease inhibitor	Q6YEY6	TC70006		x	
1612552_at	Putative S-adenosyl-L-methionine:salicylic acid carboxyl methyltransferase	Q9C9W8	TC57170		x	
1620309_at	Putative S-adenosyl-L-methionine:salicylic acid carboxyl methyltransferase	Q9C9W8	TC63451		x	
1622147_at	1-Aminocyclopropane-1-carboxylate oxidase 3	Q08507	TC60326		x	
1616358_at	MLO-like protein 11	Q9FI00	BQ798612			x
*Cell wall metabolism*						
1608074_s_at	Expansin	Q84UT0	TC62965			x
1620840_at	Alpha-expansin	Q8LKJ8	TC53122			x
1615995_at	Xyloglucan endotransglycosylase XET2	Q9LLC2	CF212592			x
1620003_at	Xyloglucan endotransglycosylase 1	Q9ZRV1	TC63269			x
1608799_at	Pectin methylesterase	Q96497	TC58800			x
1619468_at	Pectin methylesterase PME1	Q94B16	TC53043			x
1619522_at	Putative beta-galactosidase BG1	Q94B17	TC56838			x
1608756_at	Polygalacturonase-like protein	Q84LI7	TC59719			x
1606763_at	Putative beta-1,3-glucanase	Q8L868	TC67051			x
1609506_at	Putative cellulase CEL2	Q94B13	NP596365			x
1610263_at	Putative beta-1,3-glucanase	Q8L868	TC67051			x
*Cell duplication*						
1612320_a_at	Tubulin alpha chain	P33629	TC57547			x
1616815_at	Tubulin beta-8 chain	Q41785	TC55048			x
1618413_at	Tubulin alpha chain	P33629	TC63601			x
1619167_at	Tubulin beta-8 chain	Q41785	TC62643			x
1621015_at	Alpha-tubulin 1	Q8H6M1	TC65238			x
1622466_at	Tubulin beta-8 chain	Q41785	TC62809			x
1608927_at	Putative histone H2A	Q6L500	TC53574			x
1612573_at	Histone H3	A2Y533	TC56731			x
1613041_at	Histone H4	Q76H85	TC61904			x
1613076_at	Histone H4	Q76H85	TC62637			x
1620332_at	Histone H3	A2Y533	TC59489			x
1622440_at	Histone H3	A2Y533	TC64779			x
1622737_at	Histone H2B	O22582	TC64405			x
1610854_at	Proliferating cell nuclear antigen	P22177	TC54817			x
1610422_at	Patellin-6	Q9SCU1	TC61622			x
1610607_at	Gip1-like protein	Q93WR4	TC66111			x
1613373_at	Formin-like protein 1	Q8S0F0	TC55249			x
1607792_at	Putative DNA polymerase alpha catalytic subunit	O48653	TC59012			x

^a^Cluster or Affy ID of transcripts modulated at 2 or 6 h. (+) and (-) refer to up- and down-regulation in the treated sample with respect to the control.

^b^UniprotID [73]of the first hit obtained by “Blast” analysis.

^c^TC: corresponding grapevine Tentative Consensus sequence obtained by a search (BlastN) against the Grape Gene Index database [75].

At 2 h the treatment caused positive transcriptional regulation of a grapevine gene (CLU090) encoding a protein with homology to an Arabidopsis kinase-associated protein phosphatase (KAPP) (Table 1). KAPP protein may function as a signalling component in the pathway involving the serine-threonine receptor-like kinase, RLK5 of Arabidopsis [24]. In rice the RLK XA21 confers resistance to bacterial blight disease [25]. Other genes possibly involved in signal transduction showed overexpression at 6 h: a gene (1620080_at) with homology to a putative receptor-like protein kinase ARK1 of *Oryza sativa *and a gene (1611172_at) homologous to a *Glycine max *Salt Overly Sensitive gene encoding a SOS2-like protein kinase (Table 1). In *Arabidopsis thaliana *ARK genes seem to be involved in plant defence response to wounding and to bacterial infections [26], while SOS2 is a signalling kinase involved in salt tolerance response [27]. Phospholipid-derived molecules are emerging as novel second messengers in plant defence signalling and phospholipases are key enzymes for their synthesis [14,28]. In the array experiment we observed the overexpression of a putative phospholipase gene (1608981_at), which may generate lipid messengers for the signalling response (Table 1).

The activation of a signal cascade generally induces the expression of genes encoding for specific transcription factors, which in turn regulate downstream effector genes.

Two genes, upregulated at 6 h, showed homology to a hot pepper WRKY-b (1610775_s_at) and Arabidopsis WRKY11 (1611285_s_at) respectively (Table 1). WRKY proteins are plant-specific transcription factors whose expression is modulated in response to wounding, pathogen infection and abiotic stress [29]. Other classes of transcription factors appeared to take part in regulation of the response of grapevine cells to DIMEB treatment. The grape homologue (1619311_at) of a tomato pathogenesis-related gene transcriptional activator PTI5 was upregulated at 6 h (Table 1). This transcription factor binds to the GCC-box *cis *element present in the promoter region of many plant PR genes [30] and its upregulation could explain the observed induction of many PR proteins in this experiment. Another sequence (CLU059), induced at 2 h, which might modulate the expression of PR genes is the homologue of the tobacco bZIP TGA10 factor (Table 1). It has been reported that this protein can bind to the regulatory activation sequence-1 (*as-1*) [31] identified in the promoter of Arabidopsis *PR-1 *gene [32].

**Figure 2:**
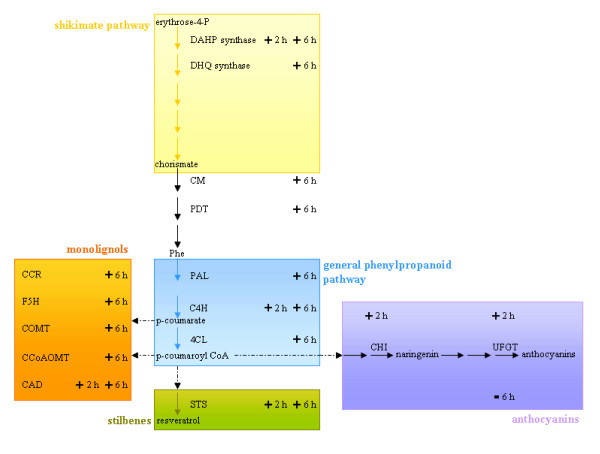
**Modulation of secondary metabolism at 2 and 6 h after DIMEB treatment**. Modulation (+ or -) of genes encoding enzymes of phenylalanine biosynthesis, general phenylpropanoid metabolism, monolignol, stilbene and anthocyanin pathways are reported within a simplified secondary metabolism scheme. Abbreviations: DHAP synthase, 3-deoxy-d-arabino-heptulosonate 7-phosphate synthase; DHQ synthase, 3-dehydroquinate synthase; CM, chorismate mutase; PDT, prephenate dehydratase; PAL, phenylalanine ammonia-lyase; C4H, cinnamate 4-hydroxylase; 4CL, 4-coumarate-CoA ligase; CAD, cinnamyl alchol dehydrogenase; CCoAOMT, caffeoyl-CoA 3-O-methyltransferase; COMT, caffeic acid O-methyltransferase; CCR, cinnamoyl-CoA reductase; F5H, ferulate-5-hydroxylase; STS, stilbene synthase; CHI, chalcone isomerase; UFGT, flavonoid-3-O-glucosyltransferase.

Our results indicated that one of the final grapevine cell responses to the DIMEB-elicited signal consists in the modulation of phenolic metabolism, especially stilbene and monolignol biosynthesis (Figure 2).

Genes encoding enzymes involved in phenylalanine biosynthesis such as 3-deoxy-d-arabino-heptulosonate 7-phosphate synthase (CLU083; 1611211_at; 1614440_at; 1619357_at; 1621405_at), 3-dehydroquinate synthase (1609646_at), prephenate dehydratase (1609932_at; 1621307_at) and chorismate mutase (1611895_at) were positively modulated both at 2 and 6 h after DIMEB treatment (Table 1). These enzymes participate in the synthesis of aromatic amino acids, particularly of phenylalanine, which is the link between primary and secondary metabolism, being a precursor of general phenylpropanoid metabolism. A recent report showed that cyclodextrins stimulates the expression of the structural genes of the general phenylpropanoids metabolism which sustains the synthesis of p-cumaroyl CoA, one of the two precursors of stilbenes [17].

Although we focused on the earlier cell response time, at both time points we also observed upregulation of this pathway's genes, namely phenylalanine ammonia lyase (1613113_at), cinnamic acid 4-hydroxylase (CLU024; 1610821_at; 1616191_s_at) and 4-coumarate-CoA ligase (1615801_at; 1619320_at) (Table 1). Similarly, several stilbene synthase genes were induced at 2 h and 6 h (CLU009, CLU022, CLU023, CLU049, CLU097, CLU103, 1606750_at, 1608009_s_at, 1609696_x_at, 1609697_at, 1610824_s_at, 1610850_at, 1611190_s_at, 1612804_at, 1614621_at, 1616575_at, 1620964_s_at, 1622638_x_at). According to the classification proposed by Richter et al. [33], they correspond to 7 different stilbene synthase genes plus one pseudogene (1606750_at). In particular, the probeset 1616575_at, encoding a stilbene synthase 2, appeared to be the most induced one, being 23 times higher in the DIMEB treated sample with respect to the control. In agreement, the chemical analysis proved stilbene accumulation in the medium already at 2 h and at higher levels after 6 h, as previously reported [21].

The accumulation of stilbenes in the growth medium requires, besides stilbene biosynthesis, the presence of export machinery. In fact, induction of genes encoding putative secondary metabolite transporters, such as those belonging to the ATP-binding cassette (ABC) transporter family, was found. Genes encoding for pleiotropic drug resistance (PDR)-like ABC transporters (CLU106; CLU119), ABC transporter-like proteins (1613763_at; 1618493_s_at) and a CjMDR transporter (1610363_at) were indeed induced (Table 1). The ABC transporters play an important role in some host-pathogen interactions [34]. In some pathogenic fungi they are involved in resistance to plant phytolexins and antifungal compounds, while in plants they seem to take part in plant defence response [34]. The induction of genes encoding glutathione S-transferase (1609330_at; 1611890_at) at 6 h correlates well with the ABC-mediated transport (Table 1). A glutathione moiety seems to function as a "recognition tag" for the transport of phenols [35]. Resveratrol translocation outside the cells has two main objectives: to mediate the defence response against pathogens and to avoid intracellular accumulation of this compound at cytotoxic levels.

Phenylpropanoid metabolism also produces the precursors (p-coumarate and p-coumaroyl-CoA) for the synthesis of monolignols, which are used to reinforce the cell wall during defence response [36]. DIMEB treatment caused a general induction of genes involved in their synthesis at 6 h: the genes for caffeic acid O-methyltransferase (1607475_s_at, 1619682_x_at, 1620342_at), caffeoyl-CoA *O*-methyltransferase (1611897_s_at; 1614643_at), cinnamyl alcohol dehydrogenase (1613900_at), ferulate 5-hydroxylase (1614045_at; 1614502_at) and cinnamoyl-CoA reductase (1619065_at) were overexpressed (Table 1, Figure 2). Genes coding for enzymes such as polyphenol oxidase and diphenol oxidase, probably responsible for the lignin polymerization process [36], were induced as well (1622651_at; 1610806_at) (Table 1).

The other branches of phenolic metabolism seemed not to be affected by DIMEB. Only two genes of the anthocyanin pathway (a chalcone-flavonone isomerase (CLU122) and a flavonol-3-O-glucosyltransferase (CLU048)) were induced at 2 h but not at 6 h (Table 1, Figure 2). Interestingly, selective induction of the early steps of phenylpropanoid metabolism and of the late steps leading to monolignol biosynthesis was also described in Arabidopsis in the early response to oligogalacturonide treatment [37].

The results strongly suggest that DIMEB acts as an elicitor modifying cell metabolism to promote the accumulation of phytoalexins and cell wall lignification. These two defence responses have been described as typical biochemical responses occurring in vegetal cells after elicitor exposure [14].

The transcriptional profiling results, however, show that the response to DIMEB seems to include other defence mechanisms. Overexpression of sequences for pathogenesis-related proteins such as chitinase (CLU088; 1613871_at; 1617192_at; 1617430_s_at), PR-10 (CLU001; 1610011_s_at; 1618568_s_at) and PR-4 (CLU021), but also for a prolin-rich protein (CLU036) and a protease inhibitor (1609875_at; 1611666_s_at) was observed in both experiments, while upregulation of two genes encoding the S-adenosyl-L-methyonine:salicylic acid carboxyl methyltransferase (1612552_at; 1620309_at) was recorded at 6 h (Table 1). Interestingly, this enzyme mediates the synthesis of gaseous methyl salicylate which was recently demonstrated to be a key mediator in plant systemic acquired resistance [38] in tobacco, as well as an inducer of the expression of *PR-1 *gene and TMV resistance [39]. This result strengthens the hypothesis that DIMEB acts as a true elicitor. The increase in the expression of a gene encoding for a 1-aminocyclopropane-1-carboxylate oxidase (1622147_at), would suggest the involvement of ethylene as well (Table 1). This hormone is a major regulator of the plant's reaction to pathogen attack [40] and via the action of a group of ethylene responsive factors it modulates the expression of plant defence-related genes such as, for example, phenylalanine ammonia-lyase, hydroxylproline-rich glycoprotein and acid class II chitinase [41,42]. It appears from the finding that a gene (1616358_at) homologous to an MLO-like 11 of Arabidopsis was downregulated at 6 h (Table 1), that the similarities between the cell's responses upon DIMEB treatment and upon pathogen attack are even greater. In barley, downregulation of the *Mlo *gene is involved in response to powdery mildew caused by the fungus *Blumeria graminis *f.sp.*hordei *[43], and in the dicot *Arabidopsis thaliana*, resistance to powdery mildews also depends on loss-of-function mlo alleles [44].

Our data support another effect of DIMEB on grapevine cells: blockage of the cell-division process. Upon treatment, we measured a lower expression of the genes involved in modification of the cell wall structure, cell division and microtubule organization. At 6 h, downregulation of genes related to cell wall modification [45], such as those encoding expansins (1608074_s_at; 1620840_at), xyloglucan endotransglycosylase (1615995_at; 1620003_at), pectin methylesterases (1608799_at; 1619468_at), a β-galactosidase (1619522_at), a polygalacturonase (1608756_at) and endoglucanases (1606763_at; 1609506_at; 1610263_at), was observed (Table 1). The sequence 1609506_at corresponds to the *VvCEL2 *transcript which encodes a grapevine cellulase. Since in Arabidopsis the expression of the cel1 gene was related to growing tissues [46], downregulation of *VvCEL2 *could be related to repression of the cell growth. Microtubules play an essential role in cell division and cell elongation too. They set the cellular division planes and axes of elongation and influence the deposition and orientation of cellulose microfibrils [47]. The downregulation of genes coding for α- and β-tubulin (1612320_a_at; 1616815_at; 1618413_at; 1619167_at; 1621015_at; 1622466_at) is indication of a stop in cell expansion and cell division (Table 1). mRNA degradation of a β-tubulin isoform was observed in soybean cells elicited by *Phytophthora sojae*-derived glucan fragments suggesting re-routing of the cellular resources towards the defence-related metabolism and repression of the cellular growth [48].

Further indication of cell division reduction were the lower transcription of genes coding for histones H2A, H3, H4 and H2B (1608927_at; 1612573_at; 1613041_at; 1613076_at; 1620332_at; 1622440_at; 1622737_at), a cyclin (1610854_at), a pattelin protein (1610422_at), a GA-induced-like protein (GIP-like) (1610607_at), a putative formin homology (FH) protein (1613373_at) and a DNA polymerase alpha catalytic subunit gene (1607792_at) (Table 1). All these proteins are either related to DNA organization and synthesis or to the cytokinesis process. The down-regulated grapevine GIP gene is homologous to GIP-5 of *Petunia hybrida*, which is expressed during the cell division phase in stems and corollas [49]. In Arabidopsis patellin1 plays a role in membrane-trafficking when the cell-plate is formed during cytokinesis [50], and formins are plant cytoskeleton-organizing proteins which take part in cytokinesis and in the establishment and maintenance of cell polarity [51]. Very similar effects on cell growth have been reported upon elicitation of parsley cell cultures with an oligopeptide elicitor. Pep 25 provoked the repression of genes regulating the cell cycle, such as cdc2, cyclin and histones [52].

A likely explanation for the repression of cell division would be the need of the cell to use, almost exclusively, the transcription system as well as the available resources to establish a defence-related metabolism.

## Conclusion

The transcriptional profiles measured at 2 h and 6 h after DIMEB treatment highlight the fact that this compound is able to induce an early and specific defence response in grapevine liquid cell cultures, supporting the hypothesis of its role as a true elicitor.

**Figure 3:**
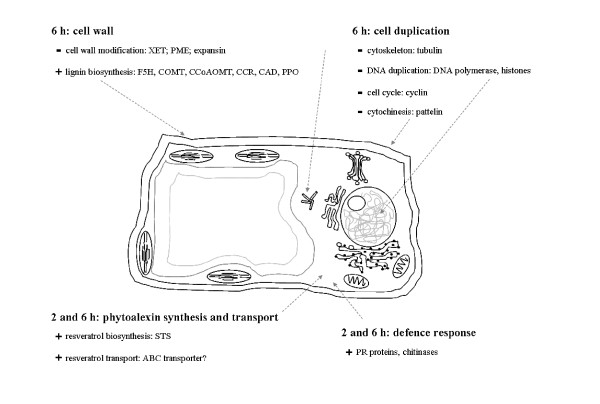
**Cellular processes triggered by DIMEB as deduced by transcriptional profiling**. Grapevine cell model showing the major genes involved in the cellular processes modulated by DIMEB treatment. Abbreviations: CAD, cinnamyl alchol dehydrogenase; CCoAOMT, caffeoyl-CoA 3-O-methyltransferase; COMT, caffeic acid O-methyltransferase; CCR, cinnamoyl-CoA reductase; F5H, ferulate-5-hydroxylase; PME, pectin methylesterase; PPO, polyphenol oxidase, PR protein, pathogenesis-related protein; STS, stilbene synthase; XET, xyloglucan endotransglycosylase.

The classes of genes modulated by the treatment reveal that DIMEB triggers a signal transduction cascade which activates different families of transcription factors, in turn modulating the effector genes of specific metabolisms. These results thus suggest that in grapevine cells DIMEB induces a stop in cell division, reinforcement of the cell wall and the production of resveratrol and defence proteins (Figure 3). This response largely resembles that occurring upon pathogen attack.

## Methods

### Plant material

Liquid cell cultures of a cross between *Vitis riparia *and *Vitis berlandieri *were used to carry out the treatment experiments with DIMEB (50 mM) [21]. Cell cultures were collected 2 h and 6 h after DIMEB treatment from control and treated samples. Cells and medium were separated by centrifugation at 12.000 ×g for 10 min at room temperature.

### Total RNA extraction

Total RNA was extracted from control and treated samples using a modified hot-borate method, as described by Moser *et al*. [53]. DNA traces were removed by DNase I treatment (Sigma-Aldrich, St.Louis, MO, USA) according to the manufacturer's procedure. RNA was isolated from one replicate for the SSH experiment (2 h) and from 3 biological replicates for the microarray experiment (6 h).

### cDNA synthesis and SSH library construction

Double-stranded cDNA was synthesized from 0.6 μg of total RNA of the control and treated samples (2 h) using the SMART™ PCR cDNA synthesis kit (Clontech Laboratories, Mountain View, CA) as recommended by the manufacturer.

Suppression subtractive hybridization (SSH) was carried out using the PCR-Select cDNA subtraction Kit (Clontech Laboratories) according to the manufacturer's procedure. The cDNA from the treated sample was used as the "tester" while the cDNA from the control sample was used as the "driver". Following hybridization, the subtracted cDNA molecules were inserted into a pCR^® ^2.1-TOPO^® ^Vector (Invitrogen, Carlsbad, CA) and then used to transform One Shot^® ^TOP10 Chemically Competent *Escherichia coli *cells (Invitrogen). Positive transformants, based on blue/white screening, were picked and arrayed in a 384-well plate containing LB medium (Sigma-Aldrich) supplemented with ampicillin (50 μg mL^-1^) and glycerol (10% v/v). The SSH cDNA library was stored at -80°C.

### Amplification of cDNA inserts and spotting on filters

The SSH library clones were cultured overnight at 37°C in a 384-well plate with LB medium and ampicillin (50 μg mL^-1^). A small aliquot (1 μl) of each liquid culture was then transferred into four 96-well plates containing PCR mix and used as template to amplify the corresponding cDNA inserts. PCR reactions (95°C for 15 min, 94°C for 45 sec, 68°C for 45 sec, 72°C for 2 min for 35 cycles, 72°C for 7 min) contained 300 nM Nested Primer PCR 1 and 300 nM Nested Primer PCR 2R (Clontech Laboratories), 0.5 U HotStartTaq DNA polymerase (Qiagen, Shanghai, China), 200 μM dNTPs, 1.5 M betain (Sigma-Aldrich) and 80 μM Cresol Red (Sigma-Aldrich). The 40 μl PCR reactions were then concentrated by overnight incubation at 37°C. The human nebulin cDNA (NM_004543) was PCR amplified in the same way to serve as a positive control. One microliter of each concentrated cDNA insert together with one microliter of a 2 ng/μl solution of amplified nebulin were transferred onto 8 × 12 cm Hybond+ nylon membranes (Amersham, GE Healthcare Bio-Sciences AB, Little Chalfont, UK) using a manual 96-pin tool. The samples were arrayed in duplicate according to a 4 × 4 grid pattern. Before and after spotting, membranes were denatured on Whatmann 3 MM paper saturated with denaturation buffer (0.5 M NaOH, 1.5 M NaCl) for 15 min. Membranes were then neutralised on Whatmann 3 MM paper saturated with neutralization buffer (1.5 M NaCl, 0.5 M Tris-HCl, pH 7.2) for 15 min, rinsed in 2× SSC, air dried and crosslinked at 80°C for 2 h.

### Target labelling

To assess whether the isolated clones were truly positive, they were hybridized with the same total RNAs used for SSH library construction. The RNAs were DIG-labelled by reverse transcription according to Vernon et al. [54] with the following modifications: 7.5 μl of PCR DNA Labelling MIX 10× (Roche, Basel, Switzerland) and 1.5 μl of 50 μM of Oligo(dT)_20 _were added to 5 μg of total RNA of each sample (tester and driver). After incubation of the two samples at 65°C for 10 min and then on ice for 2 min, a mix of 6 μl of RT Buffer 5× (Invitrogen), 3 μl of 0.1 M DTT (Invitrogen), 1.5 μl of RNase OUT (40 U/μl) (Invitrogen) and 1.5 μl of Superscript II (200 U/μl) (Invitrogen) was added to each sample. Reverse transcription was performed at 42°C for 1 h and then continued for a further hour after addition of another 1.5 μl of Superscript II (200 U/μl) (Invitrogen). The reaction was stopped by incubation at 70°C for 15 min and was followed by treatment with 1.5 μl of RNase H (2 U/μl) (Invitrogen) at 37°C for 20 min. The digoxigenin-labelled probe of the control target was synthesized by PCR amplification of a portion of human nebulin cDNA cloned in pBluescript II SK/KS (-) (Stratagene) in the presence of PCR DNA Labelling MIX 10×. PCR reaction was carried out in 50 μl using 7 ng/μl of pBluescript II SK (-) containing human nebulin cDNA as template and the primers nebulin-for 5'-CAGGAGACTATTACAGGTTT-3' and nebulin-rev 5'-ACCCATAGGCAGCTTGAGAA-3', according to the manufacturer's procedure. PCR conditions were 95°C for 15 min, 35 cycles of 94°C for 45 sec, 52°C for 45 sec, 72°C for 1 min, followed by 72°C for 7 min.

### Hybridization, washing and detection

Two filters were incubated with 20 ml of pre-hybridization solution (5× SSC, 0.1% (w/v) N-lauroylsarcosine, 0.02% (w/v) SDS, 1% (v/v) blocking solution in 1× acid maleic buffer) at 72°C for 30 min. Two different probes were prepared: the first was obtained by mixing the DIG-labelled "tester" DNA (30 μl) with the DIG-labelled human nebulin (2 μl), the second by mixing the DIG-labelled "driver" DNA (30 μl) with the DIG-labelled human nebulin (2 μl). After a short denaturation step (95°C for 3 min) the two probes were incubated separately with one filter each overnight at 68°C in hybridization solution (20 ml, 5× SSC, 0.1% (w/v) N-lauroylsarcosine, 0.02% (w/v) SDS, 1% (v/v) blocking solution in 1× acid maleic buffer). After hybridization, four high-stringency washings at 68°C for 20 min (2× SSC, 0.5% (w/v) SDS) followed by two low-stringency washings (0.2× SSC, 0.5% (w/v) SDS) at 68°C for 20 min, were carried out. Chemiluminescence was detected by 30-min exposure to Kodak^® ^BioMax Light Film (Kodak, Rochester, NY) after incubation with anti-DIG antibodies and CDP-Star, according to the manufacturer's procedure (Roche).

### Sequencing of transcripts identified by SSH

Following the screening procedure, the 289 positive clones were amplified, as described above for filter production, but without betain and Cresol Red in the PCR reaction mix. Five microliters of each PCR reaction were purified from primers and nucleotides using 1.5 μl of ExoSAP-IT™ (Amersham) at 37°C for 1 h. The reaction was stopped at 75°C for 15 min. Three nanograms for every 100 bp of amplified fragment were used for the sequencing reaction with Nested PCR Primer 1. Sequencing of 243 positively amplified clones was outsourced to the BMR Sequencing Service of C.R.I.B.I. (University of Padua, Padua, Italy) [55]. Electropherograms were analyzed with Phred [56,57] to assign a quality score and with a perl script using the UniVec Database [58] to identify any vector and adaptors sequences. Interspersed repeats and low complexity DNA sequences were identified through analysis with RepeatMasker [59]. The sequences were then organized in transcript consensus sequences (clusters) using the CAP3 DNA sequence program [60].

### Affymetrix GeneChip experiments

Total RNA of the control and treated cells after 6 h of DIMEB treatment (3 biological replicates for each type of sample) were used to hybridize 6 different GeneChip^® ^*Vitis vinifera *Genome Arrays (Affymetrix, Santa Clara, CA). Ten micrograms of total RNA for each replicate were purified as described above (Total RNA extraction), subjected to further purification using "RNeasy" columns (Qiagen) and sent to an external service (IFOM-IEO Campus for ONCOGENOMICS, Milan, Italy) for labelling and hybridization. RNA samples passed the quality check as determined by electrophoresis run on a Agilent BioAnalyzer (Agilent, Palo Alto, CA, USA). Biotin-labelling, hybridization, washing, staining and scanning procedures were performed according to the Affymetrix technical manual. Analysis of raw data was performed using the open source software of the Bioconductor project [61,62] with the statistical R programming language [63,64]. The quality of the hybridization reactions was checked using the affyPLM package. Intensity distribution of PM for each chip and the quality of the 3 biological replicates of both control and treated conditions were analyzed with the functions and plots (histogram and MA plots) of the affy package [65-67]. Background adjustment, normalization and summarization were performed using gcrma and the affy package. Data, before and after application of the gcrma algorithm [68], were compared through the graphical representation of box-plots and MA plots. Probe sets which were not expressed or were non-differentially expressed between the two conditions considered were eliminated in a filtering step based on the inter-quantile range method (IQR = 0.25) using the genefilter package. A two-class paired SAM analysis (Δ = 0.9; FDR = 13.3%) [69] was performed using the probe sets resulting from the filtering procedure in order to identify differentially expressed probe sets between the control and treated conditions. A fold-change of two was then applied.

### Functional annotation of the SSH transcripts and Affymetrix probesets

Protein sequences encoded by the SSH transcripts or by the representative sequence of each probeset as provided by the NetAffx Analysis Center [70] were predicted using a consensus generated by three different CDS predictors [71]. Blastp analyses [72] of the polypeptides obtained from the predicted CDSs were performed by searching against the UniProt database [73]. GO terms (molecular function, biological process and cellular component) [23] were linked at every consensus sequence on the basis of the results of the Blastp analysis (Additional files 1 and 2). The sequences were organized in main functional categories according to the GO term biological process (Additional files 3 and 4). In cases of non significant Blastp results (Evalue <1e-8; sequence alignment length <75% of the query polypeptide length), these were classified as "No hits found".

The SSH transcripts were deposited at the NCBI database [74] under the sequence IDs reported in the Additional file 1. Both SSH transcripts and probesets were also referred to corresponding Tentative Consensus sequences obtained by a search (BlastN) against the Grape Gene Index database [75] and to the corresponding genomic locus on Pinot Noir clone ENTAV 115 [76] (Additional files 1 and 2).

### Real-time reverse transcription (RT)-PCR

To validate the SSH and microarray data, 12 genes and 5 genes identified by SSH and GeneChip array respectively, were also analyzed by quantitative RT-PCR experiments (Additional file 5). Specific primers were designed to generate 100–200 bp PCR products (Additional file 5). The actin gene (TC45156) was used to normalize the data (actin forward: 5'-TCCTTGCCTTGCGTCATCTAT-3'; actin reverse: 5'-CACCAATCACTCTCCTGCTACAA-3') since in preliminary trials it appeared to be constantly expressed in the RNA samples subjected to gene expression analyses. For RT-PCR, total RNA from control and treated samples of the SSH experiment and from 3 biological replicates of control and treated samples of the GeneChip experiments were used. DNA traces were removed with DNase I treatment (Sigma-Aldrich) according to the manufacturer's procedure. Reverse transcription reactions and real-time RT-PCR reactions were performed using the SuperScript™ III Platinum^® ^Two-Step qRT-PCR Kit with SYBR^® ^Green (Invitrogen) according to the manufacturer's protocols with minor modification (300 nM of each primer in a final volume of 12.5 μl). PCR reactions contained 20 ng of cDNA and were replicated 3 times (technical replicates). Amplification reactions were performed with an ABI PRISM^® ^7000 Sequence Detection System (Applied Biosystems). The following thermal profile was used: 50°C for 2 min; 95°C for 10 min; 40 cycle of 95°C for 15 sec and 55°C for 1 min. Data were analysed with the ABI PRISM^® ^7000 SDS Software (Applied Biosystems). PCR reaction efficiencies were calculated with the LinRegPCR program [77]. For all the consensus sequences, the differential expression between treated and control samples was expressed as a ratio calculated with the Pfaffl equation [78]. The overall standard error of the mean normalized expression was obtained by applying the error calculation based on Taylor's series as developed for REST© software [79].

### Data Availability

All microarray expression data are available at EBI ArrayExpress under the series entry E-MEXP-2114.

## Abbreviations

DIMEB: (heptakis(2,6-di-*O*-methyl)-*β*-cyclodextrin); SSH: Suppression subtractive hybridization; cDNA: Complementary DNA; CDS: Coding Sequence; EST: Expressed Sequence Tag; GO: Gene Ontology; NCBI: National Center for Biotechnology Information; SAM: Significance Analysis of Microarrays; RT-PCR: Real time polymerase chain reaction.

## Authors' contributions

AZ made a substantial contribution to conception, data collection and interpretation and manuscript drafting. PG participated in data analysis and manuscript writing. AC contributed to sequence analysis and annotation. SP participated in data analysis and manuscript revision. RV critically revised the manuscript. CM contributed to data interpretation and manuscript writing. RV (Velasco) and FM participated in the project's design and coordination. All authors read and approved the final manuscript.

## Supplementary Material

Additional file 1 **Functional annotation of the transcripts identified by SSH**. Cluster ID, Cluster length, GenBank Accession Number at NCBI [74], NCBI Sequence ID of the corresponding genomic locus on Pinot Noir clone ENTAV 115 [76], reference Tentative Consensus sequence in Grape Gene Index [75], GO terms, Ontology type [23], UniProtID [73], description and E-value are reported for each sequence. Click here for file

Additional file 2 **Functional annotation of differentially expressed probe sets**. AffyID, Fold change, reference sequence accession numbers, NCBI Sequence ID of the corresponding genomic locus on Pinot Noir clone ENTAV 115 [76], reference Tentative Consensus sequence in Grape Gene Index [75], GO terms, Ontology type [23] and UniProtID [73] description are reported for each probe set. Click here for file

Additional file 3 **Functional category distribution of 127 transcripts modulated at 2 h**. Each transcript is grouped in a single functional category defined by Gene Ontology "Biological process" terms [23]. Number and percentage of transcripts are reported for each main category. "No hits found" refers to transcripts with no significant homology to UniProt proteins. Click here for file

Additional file 4 **Functional category distribution of 223 upregulated and 148 downregulated probe sets**. Each probe set is grouped in a single functional category defined by Gene Ontology "Biological process" terms [23]. Number and percentage of probe sets is reported for each main category. "No hits found" refers to probe sets with no significant homology to Uniprot proteins. Click here for file

Additional file 5 **Real-time RT-PCR validation of a set of genes identified in the SSH experiment or in the microarray experiment**. ClusterID or AffyID, description, RT-PCR relative expression value (treated vs. control) and sequences of forward and reverse primers are reported for each experiment. RT-PCR data for SSH validation are expressed as means ± SE of three technical replicates, while RT-PCR data for microarray validation are expressed as means ± SE of three biological replicates. Click here for file
