# Supplementary material for: Grapevine cell early activation of specific responses to DIMEB, a resveratrol elicitor
Source: BMC Genomics. 2009 Aug 6;10:363. doi: 10.1186/1471-2164-10-363 (PMC2743712; doi:10.1186/1471-2164-10-363)
Supplement: Additional file 3 — Functional category distribution of 127 transcripts modulated at 2 h. Each transcript is grouped in a single functional category defined by Gene Ontology "Biological process" terms [23]. Number and percentage of transcripts are reported for each main category. "No hits found" refers to transcripts with no significant homology to UniProt proteins. [file 1471-2164-10-363-S3.doc]

| **Functional categories** | | **Number of clusters** |
| --- | --- | --- |
|  |  |  |
| **Biological process ND; GO:0008150** | | **8 (6.30%)** |
|  |  |  |
| **Establishment of localization; GO: 0051234** | | **4(3.15%)** |
| Transport; GO:0006810 | | 4 |
|  |  |  |
| **Cellular process; GO:0009987** |  | **6 (4.72%)** |
| Cellular component organization and biogenesis; GO:0016043 | | 4 |
| Cell comunication; GO:0007154 | | 1 |
| Signal transduction; GO:0007165 | | 1 |
| Cell homeostatis; GO:0019725 | | 1 |
|  |  |  |
| **Metabolic process; GO:0008152** | | **44 (34.64%)** |
| Carbon utilization; GO:0015976 | | 1 |
| Cellular metabolic process; GO:0044237 | | 1 |
| Generation of precursor metabolites and energy; GO:0006091 | | 5 |
| Nitrogen compound metabolic process; GO:0006807 | | 1 |
| Primary metabolic process; GO:0044238 | | 21 |
| Regulation of metabolic process; GO:0019222 | | 4 |
| Secondary metabolic process; GO:0019748 | | 11 |
|  |  |  |
| **Response to stimulus; GO:0050896** |  | **9 (7.09%)** |
| Defence response; GO:0006952 | | 6 |
| Response to endogenus stimulus: GO:0009719 | | 1 |
| Response to hormone stimulus; GO:0009725 | | 1 |
| Responce to biotic stimulus; GO:0009607 | | 3 |
| Response to other organism; GO:0051707 | | 1 |
| Response to unfolded protein; GO:0006986 | | 1 |
|  |  |  |
| **No hits found** |  | **56 (44.10%)** |
