# Supplementary material for: Grapevine cell early activation of specific responses to DIMEB, a resveratrol elicitor
Source: BMC Genomics. 2009 Aug 6;10:363. doi: 10.1186/1471-2164-10-363 (PMC2743712; doi:10.1186/1471-2164-10-363)
Supplement: Additional file 4 — Functional category distribution of 223 upregulated and 148 downregulated probe sets. Each probe set is grouped in a single functional category defined by Gene Ontology "Biological process" terms [23]. Number and percentage of probe sets is reported for each main category. "No hits found" refers to probe sets with no significant homology to Uniprot proteins. [file 1471-2164-10-363-S4.doc]

| **Functional categories** | | **Upregulated** | **Downregulated** |
| --- | --- | --- | --- |
|  |  |  |  |
| **Biological process; GO:0008150** |  | **13 (5.83%)** | **23 (15.54%)** |
|  |  |  |  |
| **Cellular process; GO:0009987** |  | **4 (1.79%)** | **23 (15.54%)** |
| Cellular adhesion; GO:0007155 | |  | 1 |
| Cellular component organization and biogenesis; GO:0016043 | | 1 | 21 |
| Cell cycle process; GO:0022402 | |  | 1 |
| Cell homeostatis; GO:0019725 | | 3 |  |
|  |  |  |  |
| **Developmental process; GO:0032502** |  | **2 (0.9%)** | **1 (0.68%)** |
|  |  |  |  |
| **Establishment of localization; GO: 0051234** | | **13 (5.83%)** | **7 (4.72%)** |
| Transport; GO:0006810 | | 13 | 7 |
|  |  |  |  |
| **Metabolic process; GO:0008152** | | **87 (39.01%)** | **25 (16.90%)** |
| Catabolic process; GO:0009056 | |  | 2 |
| Cellular metabolic process; GO:0044237 | | 6 |  |
| Generation of precursor metabolites and energy; GO:0006091 | | 7 | 1 |
| Primary metabolic process; GO:0044238 | | 32 | 19 |
| Regulation of metabolic process; GO:0019222 | | 8 | 2 |
| Secondary metabolic process; GO:0019748 | | 34 | 1 |
|  |  |  |  |
| **Multi-organism process; GO:0051704** |  | **1 (0.45%)** |  |
|  |  |  |  |
| **Response to stimulus; GO:0050896** |  | **15 (6.73%)** | **5 (3.38%)** |
| Defence response; GO:0006952 | | 7 | 3 |
| Response to stess; GO:0006950 | | 5 |  |
| Response to oxidative stress; GO:0006979 | | 3 | 1 |
| Response to wounding; GO:0009611 | | 2 |  |
| Response to endogenus stimulus: GO:0009719 | |  |  |
| Response to hormone stimulus; GO:0009725 | |  | 1 |
| Responce to biotic stimulus; GO:0009607 | | 3 |  |
| Response to other organism; GO:0051707 | | 3 |  |
|  |  |  |  |
|  |  |  |  |
| **No hits found** |  | **88 (39.46%)** | **64 (43.24%)** |
